# The Suprachiasmatic Nucleus and the Intergeniculate Leaflet of the Flat-Faced Fruit-Eating Bat (*Artibeus planirostris*): Retinal Projections and Neurochemical Anatomy

**DOI:** 10.3389/fnana.2018.00036

**Published:** 2018-05-15

**Authors:** Nelyane N. M. Santana, Marília A. S. Barros, Helder H. A. Medeiros, Melquisedec A. D. Santana, Lara L. Silva, Paulo L. A. G. Morais, Fernando V. L. Ladd, Jeferson S. Cavalcante, Ruthnaldo R. M. Lima, Judney C. Cavalcante, Miriam S. M. O. Costa, Rovena C. J. G. Engelberth, Expedito S. Nascimento Jr.

**Affiliations:** ^1^Laboratory of Neuroanatomy, Department of Morphology, Biosciences Center, Federal University of Rio Grande do Norte, Natal, Brazil; ^2^Department of Zoology, Federal University of Pernambuco, Recife, Brazil; ^3^Laboratory of Neurochemical Studies, Department of Physiology, Biosciences Center, Federal University of Rio Grande do Norte, Natal, Brazil

**Keywords:** suprachiasmatic nucleus, intergeniculate leaflet, Circadian timing system, retinal projections, neurochemical content

## Abstract

In mammals, the suprachiasmatic nucleus (SCN) and the intergeniculate leaflet (IGL) are the main components of the circadian timing system. The SCN, classically known as the master circadian clock, generates rhythms and synchronizes them to environmental cues. The IGL is a key structure that modulates SCN activity. Strategies on the use of time by animals can provide important clues about how some species are adapted to competitive process in nature. Few studies have provided information about temporal niche in bats with special attention on the neural substrate underlies circadian rhythms. The aim of this study was to investigate these circadian centers with respect to their cytoarchitecture, chemical content and retinal projections in the flat-faced fruit-eating bat (*Artibeus planirostris*), a chiropteran endemic to South America. Unlike other species of phyllostomid bats, the flat-faced fruit-eating bat’s peak of activity occurs 5 h after sunset. This raises several questions about the structure and function of the SCN and IGL in this species. We carried out a mapping of the retinal projections and cytoarchitectural study of the nuclei using qualitative and quantitative approaches. Based on relative optical density findings, the SCN and IGL of the flat-faced fruit-eating bat receive bilaterally symmetric retinal innervation. The SCN contains vasopressin (VP) and vasoactive intestinal polypeptide (VIP) neurons with neuropeptide Y (NPY), serotonin (5-HT) and glutamic acid decarboxylase (GAD) immunopositive fibers/terminals and is marked by intense glial fibrillary acidic protein (GFAP) immunoreactivity. The IGL contains NPY perikarya as well as GAD and 5-HT immunopositive terminals and is characterized by dense GFAP immunostaining. In addition, stereological tools were combined with Nissl stained sections to estimate the volumes of the circadian centers. Taken together, the present results in the flat-faced fruit-eating bat reveal some differences compared to other bat species which might explain the divergence in the hourly activity among bats in order to reduce the competitive potential and resource partitioning in nature.

## Introduction

Biological rhythms are ubiquitous in nature and occurs in all living organisms (Foster and Kreitzman, 2014). In mammalians, the CTS orchestrates body rhythms for concerted actions and entrains them to photoperiodic or non-photic stimuli (Kronfeld-Schor et al., 2013). This system is built from a neural network of oscillators, modulating structures, synchronizing pathways, and efferent projections (Morin, 2013) and a molecular machinery formed by CCG, proteins and the transcriptional-translational feedback loops (Reppert and Weaver, 2001; Albrecht, 2012).

The SCN of the hypothalamus is the central circadian pacemaker (Moore and Lenn, 1972). This structure is a paired nucleus located in the anteroventral hypothalamus, on each side of the third ventricle, immediately dorsal to the optic chiasm (Van den Pol, 1991). Previous studies have shown that the SCN can be divided in two zones, a ventrolateral “core” region and a dorsomedial “shell” region, on the basis of the neuronal cytoarchitecture (Van den Pol, 1980), neurochemical phenotype (Moore et al., 2002; Morin, 2013; Allali et al., 2017), organization of afferent innervation (Moga and Moore, 1997), distribution of efferent projections (Leak and Moore, 2001), pattern of gene expression (Dardente et al., 2002) and electrical activity (Schaap et al., 2003).

The core predominantly contains VIP neurons and receives dense retinal afferents originating from melanopsin-expressing ipRGCs (Baver et al., 2008; Paul et al., 2009). Furthermore, the core is innervated by the geniculo-hypothalamic tract, consisting of axonal projections of NPY neurons from the IGL. In contrast, the shell is sparsely innervated by retinal afferents and contains vasopressin (VP)-producing cells. This portion receives and possibly integrates inputs to the core (Morin et al., 2006) and other regions of the brain (Moga and Moore, 1997). The integration of these afferents by the shell can provide a precise temporal signal to physiological and behavioral effectors (Moga and Moore, 1997). The neurochemical composition of the master clock has an impressive relevance on function, as well as molecular process underlying the synchronization of the SCN by environmental cues. It is well known that in mammals, the light-dark cycle (LD) is the strongest Zeitgeber of the master clock inducing alternating activation of the gene expression by different proteins. These rhythmic self-regulating proteins are controlled by CCG inside the SCN (Takahashi et al., 2008; Mohawk et al., 2012).

Besides retina, the principal modulating structure of the SCN is the IGL, a thin and elongated retinorecipient cell layer, which, in non-primate species, is intercalated between the dorsal (DLG) and ventral (VLG) lateral geniculate nuclei. The IGL is not directly involved in photic synchronization (Klein and Moore, 1979). It is postulated that the IGL conveys photic information to the SCN (Aronin et al., 1990) and integrates the photic and non-photic stimuli necessary to modify SCN function (Janik and Mrosovsky, 1994).

Over the course of evolution, organisms have had to adapt to photoperiodic changes, restricting many of their physiological and behavioral activities to specific phases of the light-dark cycle (Kronfeld-Schor et al., 2013). Nocturnal species developed perceptive senses of smell and hearing and concentrate their activity in the dark phase of the cycle (Sousa and Menezes, 2006). Some animals, such as bats, have developed additional specializations (echolocation and flying), which allow them to exploit the environment and forage for food (Simmons, 1989; Scalia et al., 2015). Chiropterans are exclusively or almost exclusively nocturnal (Rydell and Speakman, 1995), and their daily patterns of activity and behavior are influenced principally by components of the light/dark cycle, such as dusk, dawn (Erket, 1982), light intensity (Haeussler and Erkert, 1978), moonlight (Apple et al., 2017) and night length (Frafjord, 2013). Understandably, despite a lack of neuroanatomic and quantitative information, there is great interest in the analysis of the activity patterns of these animals in relation to chronobiology (Koilraj et al., 2000) and ecology (Ortêncio-Filho et al., 2010).

Ecological studies of the neotropical Phyllostomidade family have described a variety of activity patterns, such as bimodal and unimodal peaks of activity (Weinbeer et al., 2006). Bats in the sub-family Carolliinae express the greatest activity 2 h after the sunset, with uniform activity for the rest of the night (Bernard, 2002). However, a second activity peak during the 5th and 6th hour after sunset was described for the short-tailed fruit bat (*Carollia perspicillata*) (Pedro and Taddei, 2002), with decreasing activity for the remainder of the night (Aguiar and Marinho-Filho, 2004; Ortêncio-Filho et al., 2010). In the present study, we describe the retinal afferents to the SCN and IGL, as well as the neurochemical signatures of both nuclei, in the flat-faced fruit-eating bat (*Artibeus planirostris*). In contrast to other bats in the same genus (Ramirez-Pulido and Armella, 1987; Aguiar and Marinho-Filho, 2004), the peak of activity in *A. planirostris* occurs 5 h after sunset, followed by decreased levels of activity for the remaining hours (Marinho-Filho and Sazima, 1989). Although time is not exactly a resource as water or food, activity pattern has been considered one of the most important niche dimensions. Clearly, the patterns of nocturnality among bats are diverse (Ortêncio-Filho et al., 2010) and studies of neuroanatomical circadian nuclei in bats would be helpful to understand how slight structural differences can produce diversity in output behaviors.

## Materials and Methods

### Animals

Thirteen adult male flat-faced fruit-eating bats (39.5 – 47 g) were captured in a green urban area of the Federal University of Rio Grande do Norte, Natal, Rio Grande do Norte, Northeastern Brazil. The capture and collection of the animals were authorized by the Chico Mendes Institute for Biodiversity Conservation (Permit SISBIO/ICMBio #25233-2). Approval for the experiments was obtained from the local Animal Experimentation Ethics Committee (Protocol #009/2012). Animals were maintained at 22°C, 50% humidity in a 12:12 h LD cycle. Food (fruits) and water were available *ad libitum*. The experimental procedures were in accordance with the National Research Council Guidelines for the Care and Use of Mammals in Neuroscience and Behavioral Research.

### Intraocular Tracer Injection

Bats were anesthetized with a pharmacological mixture of tramadol hydrochloride (5 mg/kg i.m.), ketamine (5 mg/kg i.m.), diazepam (0.5 mg/kg i.m.) and xylazine (0.5 mg/kg i.m). While maintained at the appropriate level of anesthesia, the animals received, via needle attached to a micropump, 15-μl injections of unconjugated CTb (List Biological Laboratories, Campbell, CA, United States) in one eye.

The monocular injections were administered into the vitreous humor of the left eye and contained 1% CTb mixed with 5% dimethyl sulfoxide. Five days post-injection, the bats were anesthetized with the same anesthetic protocol as the surgical procedure and subjected to the following procedures.

### Perfusions and Immunohistochemistry

All animals were sacrificed by transcardiac perfusion with 150 ml of phosphate-buffered saline (PBS), pH 7.4, followed by 300 ml of 4% paraformaldehyde (Dinâmica, Diadema, São Paulo, Brazil) in PBS. After removal, the brains were postfixed in the same fixative for 2–4 h then stored in a 30% sucrose in PB solution for cryoprotection prior to slicing. Frozen brains were cut into 30-μm coronal sections. Slices with SCN were collected from 0.45 mm to 1.08 mm post-bregma, approximately at the same level of the anterior commissure in the rostral section. On the other hand, slices containing IGL were collected from 3.84 mm to 5.04 mm post-bregma, approximately at same level of the external medullary lamina in the rostral level. All sections were collected in PB, and a one-in-six series was processed for CTb. Floating sections were incubated with goat anti-CTb antiserum (List Biological Laboratories, Campbell, CA, United States) in PB containing 0.4% Triton X-100 and 5% normal donkey serum for 18–24 h. The sections were incubated with a secondary antiserum (biotinylated donkey anti-goat, Jackson Immunoresearch Laboratories, West Grove, PA, United States) in the same medium as above for 90 min.

After processing with avidin-biotin horseradish peroxidase (1:1000; Vector Laboratories, Burlingame, CA, United States) in PBS for 90 min, immunoreactivity was revealed using diaminobenzidine (DAB) and stable peroxide buffer (0.01% H_2_O_2_ in PB). The sections were mounted on pre-cleaned and chrome alum-gelatin coated slides and allowed to dry, followed by osmium tetroxide treatment. The sections were then dehydrated, delipidated and cover slipped with DPX. One of the remaining series was Nissl-stained to identify the cytoarchitectural boundaries of the regions under examination. The remaining series were immunostained for VP, VIP, NPY, 5-HT, GAD, and GFAP. The samples were incubated with their respective primary antibodies (see **Table 1**) for 18–24 h and secondary antibodies (see **Table 1**). The ABC protocol was used for immunodetection, and subsequent procedures were performed as described for CTb. Each of the remaining series was immunostained for one out of four of the designated neurochemicals, such that each antigen was studied in four animals on average. Approximately, 10 sections per animal containing the region of interest were examined. Specificity tests were based on the omission of the primary or secondary antibodies in some sections. In all cases, the immunolabelling was completely abolished.

**Table 1:** Characteristics of antibodies used.

Antibodies	Immunogen	Manufacturer, catalog and RRID	Dilution
Goat anti-CTb	Purified cholera toxin B subunit from *Vibrio cholerae* type inaba 569B	List Biological Laboratories, #CA 703, AB_10013220, polyclonal	1:1000
Guinea pig anti-(Arg8)VP	Synthetic (Arg8)Vasopressin	Península Laboratories, #CA T5048.0050, AB_2313978, polyclonal	1:1000
Mouse anti-GAD	Derived from the GAD-6-hybroma	Sigma-Aldrich, #CA G1166, AB_259846, monoclonal	1:1000
Mouse anti-GFAP	Purified GFAP from pig spinal cord	Sigma-Aldrich, #CA G3893, AB_477010, monoclonal	1:1000
Rabbit anti-5-HT	Derived from 5-HT creatinine sulfate complex conjugated to BSA	Sigma-Aldrich, #CA 5545, AB_477522, polyclonal	1:5000
Rabbit anti-NPY	synthetic NPY (porcine) conjugated to KLH	Sigma-Aldrich, #CA N9528, AB_260814, polyclonal	1:8000
Rabbit anti-VIP	Synthetic VIP	Península Laboratories, #CA T4246.0050, AB_518682, polyclonal	1:5000


### Image Analysis

Immunostained sections were examined under bright field microscopy with a Nikon Eclipse Ni microscope. Photomicrographs were obtained with a digital video camera (Nikon DS-Ri1) and were minimally processed for brightness and contrast using Canvas 12 software. Three current atlases were consulted for the identification of brain nuclei in the bat (Paxinos and Watson, 2007; Franklin and Paxinos, 2008; Scalia et al., 2013).

To quantify CTb immunoreactivity, three sections representing SCN and four sections of the IGL from five brains were analyzed bilaterally using ImageJ 1.48v software, which performed optical density (OD) measurements based on gray levels of pixels. OD has been used in previous reports to quantify similar immunostaining in these circadian centers (Saderi et al., 2013; Engelberth et al., 2014). For these measurements, the background was subtracted from the positive staining.

### Stereological Methods

The SCN and IGL volumes were estimated by Cavalieri’s method (Cavalieri, 1635; Gundersen and Jensen, 1987). This method estimates the volume of a compact object from area measurements in serial sections (Kinderlen and Dorph-Petersen, 2017). The sections were analyzed bilaterally throughout the rostrocaudal extent using Stereo investigator 11 software (Microbrightfield). The equidistant coronal section samples were obtained by a uniform, systematic and random regime (SURS) (Gundersen et al., 1999).

The Cavalieri estimator uses the following formula: *V* = *t* × (*a*/*p*) × ∑*p* × *F*^-1^, where ‘*t*’ is the section thickness, ‘*a*/*p*’ is the representative area associated with each point on the test system, ‘Σ*P*’ is the total number of points hitting the region of interest, and *F*^-1^ is the inverse of the fraction sampled (Howard and Reed, 2010). For SCN, the counting grid had ‘*a*/*p*’ = 4225 μm^2^ and for IGL, the ‘*a*/*p*’ = 1600 μm^2^. For both structures, *F*^-1^ was equal to 6.

The coefficient of error (CE) was estimated according to Gundersen et al. (1999). The mean CE volumes for SCN and IGL were 3.53 and 3.82%, respectively. To measure the cellular area of SCN and IGL neurons, NIS Elements AR 4.20 software (Nikon) was used.

### Statistical Analysis

All quantitative data are expressed as the mean ± standard deviation and were analyzed by a general linear mixed effect model (Tu, 2014), followed by Bonferroni *post hoc* tests. OD was the dependent variable; animals, section levels and antimers were designated fixed effects; and individual anatomic variability was included as a random effect. In all analyses, differences were considered significant at *p* < 0.05. Data analyses were performed using SPSS (IBM Corp) for Windows version 20.

## Results

### Suprachiasmatic Nucleus

#### Cytoarchitecture

**FIGURE 1:**
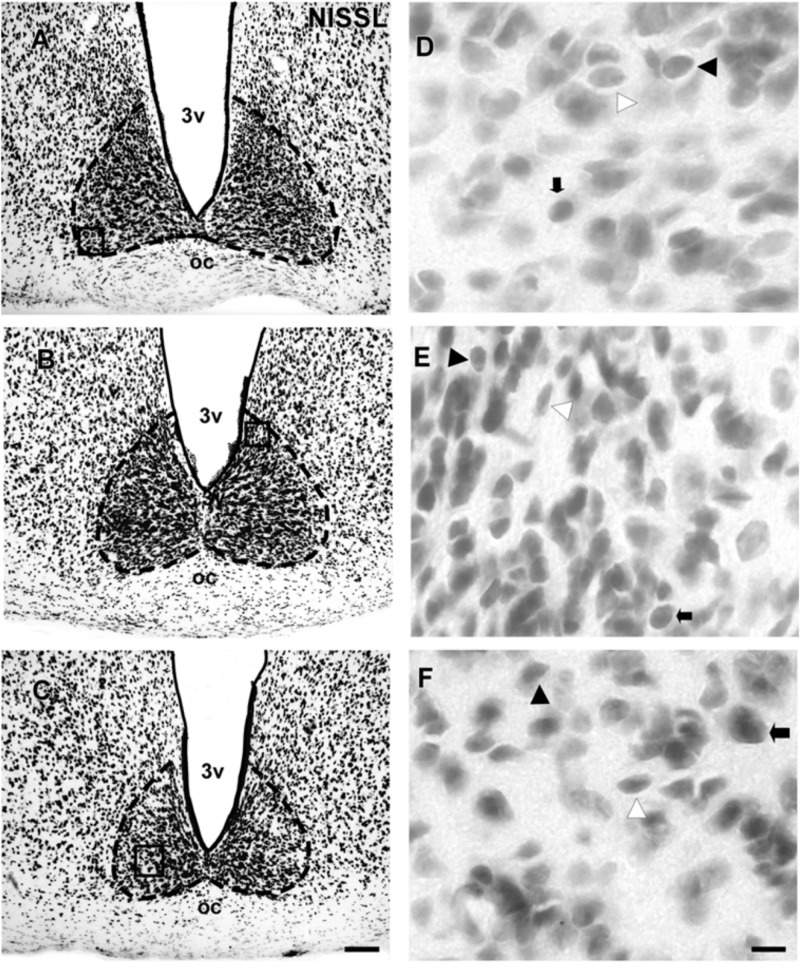
Photomicrographs of the brain sections of flat-faced fruit-eating bat showing the SCN in bright field **(A–C)** stained by Nissl technique at rostral, middle and caudal levels, respectively. The boxed areas in **(A–C)** are shown in high magnification in **(D–F)**, respectively. Black arrowheads point to oval neurons. White arrowhead points to elliptical cells. Black arrow points to spherical neurons. Scale bar 100 μm **(A–C)** and 10 μm **(D–F)**.

The SCN of the flat-faced fruit-eating bat was identified as a paired cluster of intensely stained spherical, oval, and elliptical-shaped cells (51 ± 18.65 μm^2^), located in the anterior hypothalamus, bilateral to the third ventricle and dorsal to the optic chiasm. At the rostral level, the SCN exhibited an approximately triangular shape, and at the mid- and caudal levels, this structure assumed a pear-shaped contour, with its larger axis directed dorsoventrally (**Figure 1**). At all rostro-caudal levels there was an agglomerate of compact and darkly stained cells in the core region, surrounded by an area of more sparsely distributed and less intensely stained cells. The estimated mean volume of the SCN was 0.1041 mm^3^ (**Table 2**).

**Table 2:** Comparative analysis of morphometric and stereological aspects of the SCN and IGL from mammalians.

Animal	Strain/Species	SCN		
		
		Length (mm)	Volume (mm^3^)	Reference
Rats	Fischer	0.9	–	Van den Pol, 1980
	Sprague-Dawley	0.73	0.036 0.064	Moore et al., 2002 Güldner, 1983
	Long Evans	–	0.054	Bloch and Gorski, 1988
		≈0.8	–	Pinato et al., 2007
	Wistar	≈0.8	0.044	Pinato et al., 2007
Hamster	*Mesocricetus auratus*	0.6	–	Lydic et al., 1982
Cat	*Felix domesticus*	0.9	–	Lydic et al., 1982
Bat	*Artibeus planirostris*	≈0.6	0.1041	Present study
Sheep	*–*	2.8–3.1	0.391–0.426	Tessonneaud et al., 1994
Primates	Marmoset (*Callithrix jacchus*)	≈0.9	–	Pinato et al., 2007
	Capuchin monkey (*Cebus apella*)	≈1.05	0.226	Pinato et al., 2007
	Rhesus (*Macaca mulatta*)	0.9	–	Lydic et al., 1982
	Human	1.47	0.267–0.464	Swaab and Hofman, 1990
		–	2.2	Moore, 1993
Tree shrew	*Tupaia belangeri chinensis*	0.7	–	Ni et al., 2014

**Animal**	**Strain/Species**	**IGL**		
		
		**Length (mm)**	**Volume (mm^3^)**	**Reference**

Rats	Sprague-Dawley	≈2	–	Moore and Card, 1994
Hamster	*Mesocricetus auratus*	≈2.2	–	Morin et al., 1992
Bat	*Artibeus planirostris*	≈0.6	0.0378	Present study
Tree shrew	*Tupaia belangeri*	0.05–0.2	–	Conley and Friederich-Ecsy, 1993


#### Retinal Projections

**FIGURE 2:**
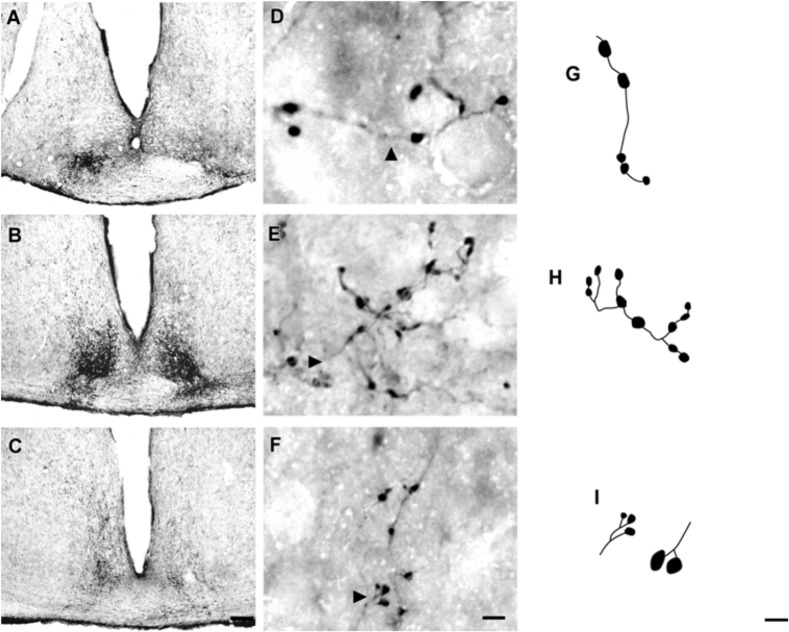
Photomicrographs of the SCN coronal sections of flat-faced fruit-eating bat at rostral **(A)**, middle **(B)** and caudal **(C)** levels, illustrating the distribution pattern of retinal projections in the ipsi and contralateral sides. The boxed areas in **(A–C)** are shown in high magnification in **(D–F)**, respectively, illustrating the detailed morphology of the retinal axons in the SCN showing cholera toxin-labeled varicosities in the simple endings **(D)**, string-like terminals **(E)** and R2-like terminals **(F)**. Black arrowheads point to sort of fibers. **(G–I)** Camera-lucida drawings that depict the morphology of simple endings **(G)**; string-like terminals **(H)** and R2-like terminals **(I)**. Scale bar 100 μm **(A–C)** and 10 μm **(D–I)**.

At the rostral levels, CTb-IR fibers were contralateral in the SCN. At the remained levels, CTb-IR fibers were evident bilaterally in the SCN of the flat-faced fruit-eating bat. Between the ipsilateral (0.9981 ± 0.0001) and contralateral (0.9981 ± 0.00009) sides, there was no significant difference (*p* = 0.3) in CTb-IR fibers assessed by OD in the SCN as a whole. Rostrally, CTb-IR terminals were located in the ventral portion of the SCN (**Figure 2A**). At the middle level, CTb-IR fibers were more concentrated in the ventrolateral region, avoiding the laterodorsal part of the nucleus (**Figure 2B**). In the caudal sections, the CTb-IR terminals were restricted to the central region of the SCN (**Figure 2C**).

Adopting the criteria and nomenclature of Ling et al. (1997) and Morais et al. (2014) for retinothalamic fiber morphology, we identified three groups of labeled axons based on axon branching patterns and size, morphology, and complexity of bouton arrangement. The retinal fibers in the SCN could be classified as (1) simple *en passant* varicosities and terminal swellings; these sort of terminals are present in poorly branched fibers decorated with varicosities and swellings of various sizes, with a simple terminal bouton at the end of each branch of the terminal arbor (**Figures 2D,G**); (2) string-like configurations that comprised axon collaterals studded with boutons of various sizes; these swellings occur close together, and beaded collaterals form a longitudinal arrangement in which at least two fibers travel together for a distance (**Figures 2E,H**); and (3) type R2-like terminals (see Ling et al., 1997) consisting of rosette-like clusters of boutons of medium and small varicosities that emerge from the fine axon (**Figures 2F,I**).

#### Neurochemical Characteristics

**FIGURE 3:**
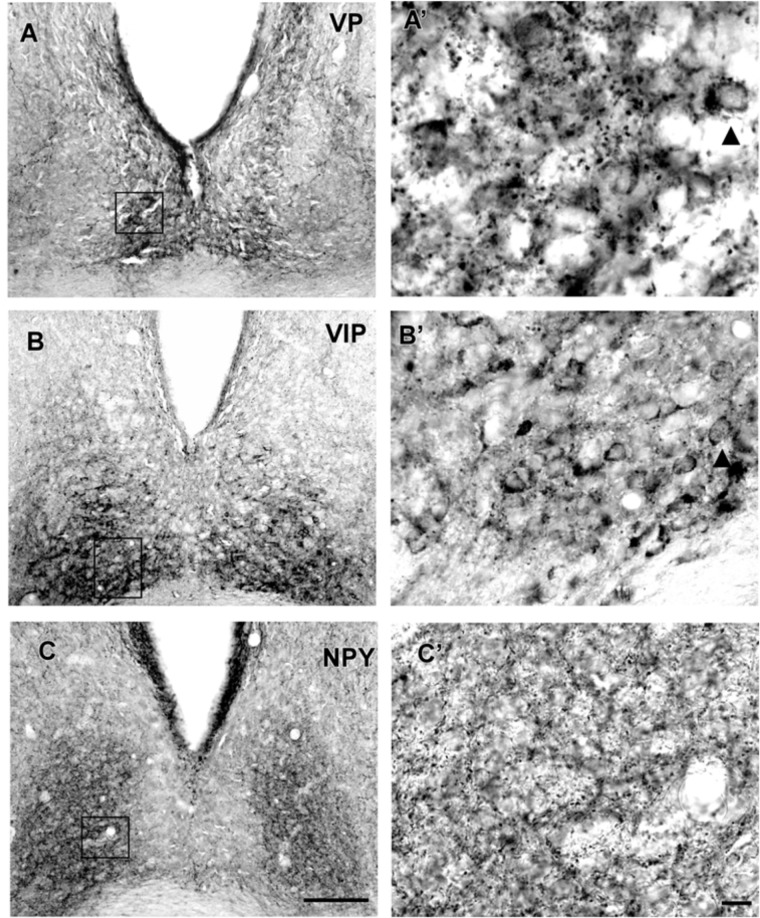
Photomicrographs of SCN coronal sections showing the immunoreactivity pattern against VP at caudal level **(A,A’)**, VIP **(B,B’),** and NPY **(C,C’)** at the middle level. Black arrowheads point to spherical VP-IR and oval VIP-IR neurons. oc, optic chiasm; 3v, third ventricle; SCN, suprachiasmatic nucleus. Scale bar 100 μm **(A,B)** and 10 μm **(A’,B’)**.

**FIGURE 4:**
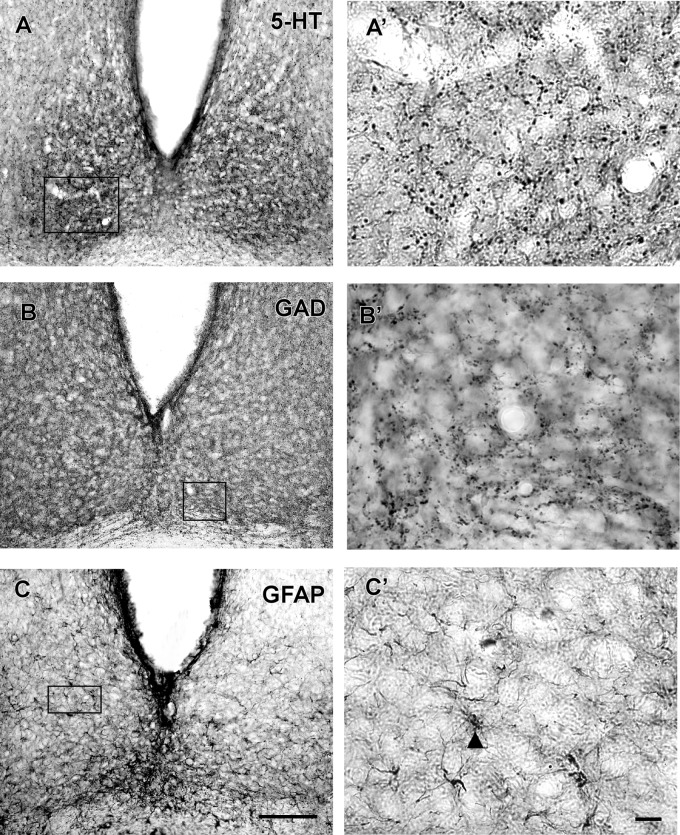
Photomicrographs of SCN coronal sections showing the immunoreactivity pattern against 5-HT **(A,A’)**, GAD **(B,B’)** at the middle level, and GFAP at the middle level **(C,C’)**. Black arrowheads point to GFAP-IR cells. oc, optic chiasm; 3v, third ventricle; SCN, suprachiasmatic nucleus. Scale bar 100 μm **(A–C)** and 10 μm **(A’–C’)**.

**FIGURE 5:**
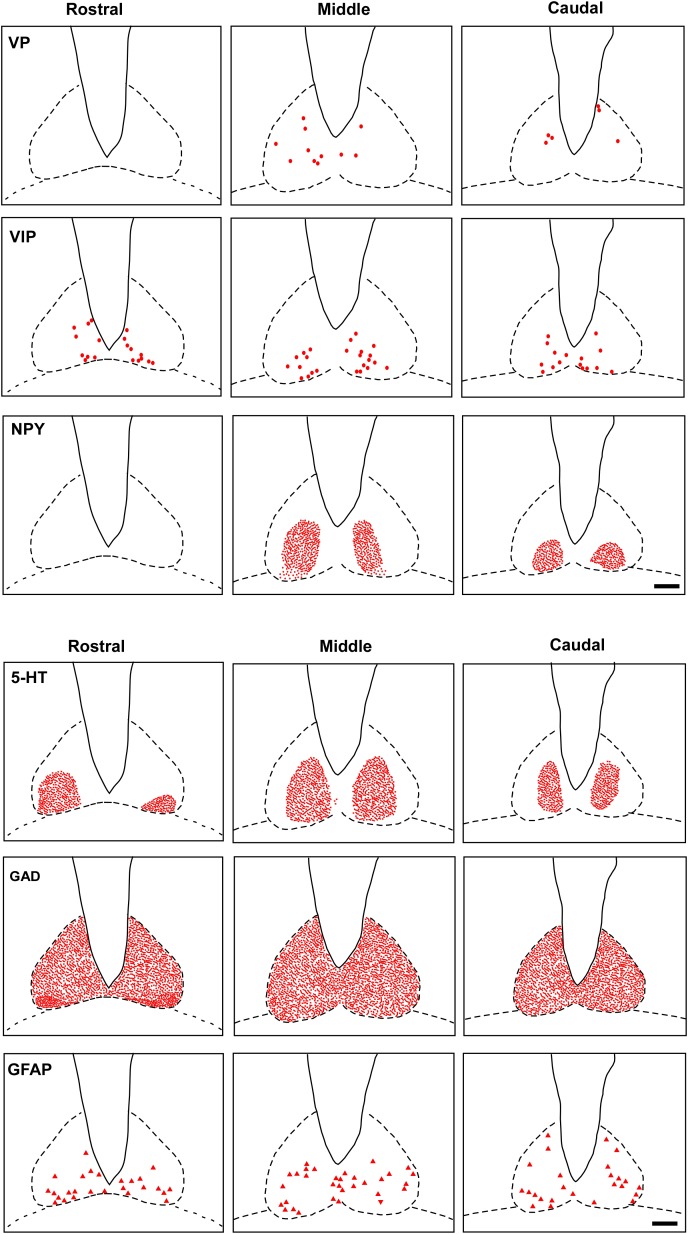
Schematic representation of the distribution of VIP, VP, NPY, 5-HT, GAD, GFAP cells and fibers-IR at three rostrocaudal levels through the SCN. Dots represent perikarya. Square and line represent fibers and terminals. Triangle represents astrocytes. oc, optic chiasm; 3v, third ventricle; SCN, suprachiasmatic nucleus. Scale bar 100 μm.

VP-IR neurons were restricted to the middle and caudal levels of the SCN. Spherical parvocellular neurons were observed immersed in a moderate neuropil, being more concentrated in the dorsomedial portion (**Figures 3A,A’**, **5**). VIP-IR perikarya were detected throughout the rostrocaudal extent of the SCN. Oval neurons immersed in a dense neuropil were identified mainly in a ventrolateral position in the SCN (**Figures 3B,B’**, **5**). Immunostaining for NPY revealed a dense network of NPY-IR fibers/terminals restricted to the ventrolateral portion of the nucleus without any specific orientation. No NPY-IR perikarya were detected in the SCN (**Figures 3C,C’**, **5**). A plexus of 5-HT-IR fibers/terminals with varicosities was found in the SCN. Rostrally, serotonergic fibers were predominantly distributed in the ventrolateral portion. At the mid-sections, 5-HT-IR fibers were located in the ventromedial position, and finally, at the caudal level, they were concentrated in the central part of the nucleus (**Figures 4A,A’**, **5**). GAD immunoreactivity revealed a moderate plexus of labeled fibers and terminals with varicosities (**Figures 4B,B’**, **5**). Immunoreactivity for GFAP (GFAP-IR) was found in the processes, as well as the cell bodies, of the astrocytes present in the SCN. This staining was evident throughout the rostrocaudal extent of the nucleus. GFAP-IR was more dense within the SCN in contrast to the surrounding hypothalamic areas (**Figures 4C,C’**, **5**).

### Intergeniculate Leaflet

**FIGURE 6:**
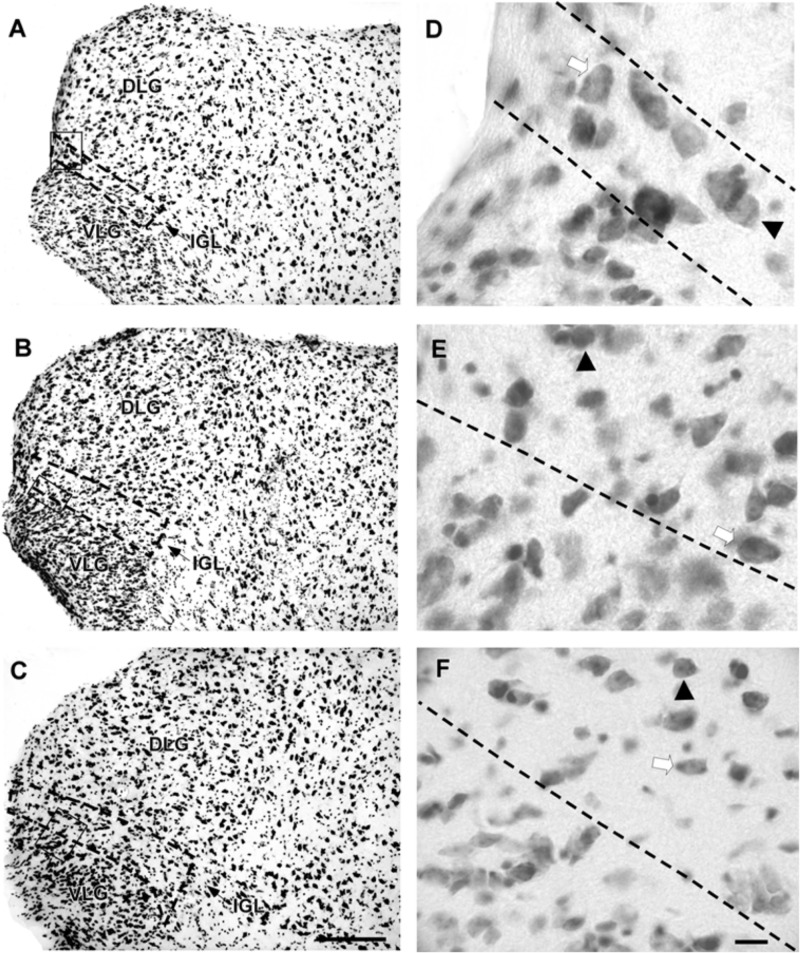
Photomicrographs of the brain sections of flat-faced fruit-eating bat showing the components of the lateral geniculate complex in bright field **(A–C)** IGL stained by Nissl technique at rostral, middle, and caudal levels, respectively. The boxed areas in **(A–C)** are shown in high magnification in **(D–F)**, respectively. Black arrowheads point to spherical neurons. White arrow points to oval cells. Scale bar 100 μm **(A–C)** and 10 μm **(E,F)**.

The lateral geniculate complex of the flat-faced fruit-eating bat appeared to contain two distinct structures: the DLG and the VLG. Less distinctly, the IGL appeared as a thin lamina of oval and spherical cells (108 ± 40.03 μm^2^) interposed between the DLG and VLG (**Figure 6**). By Cavalieri’s principle, this structure had an estimated mean volume 0.0378 mm^3^ (**Table 2**).

#### Retinal Afferents

**FIGURE 7:**
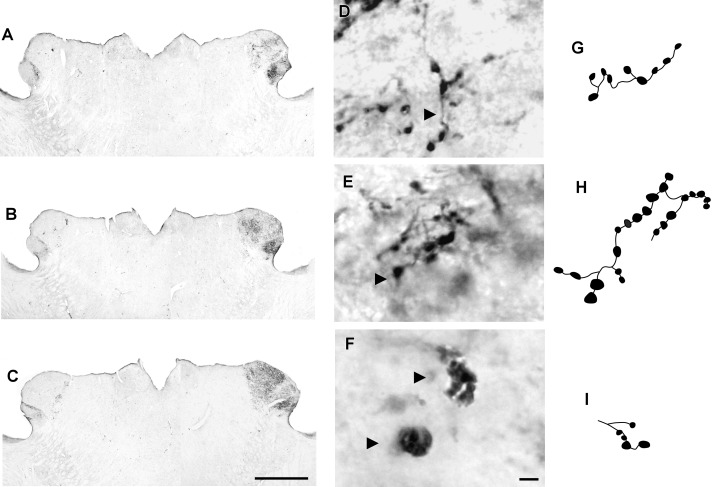
Photomicrographs of the lateral geniculate complex coronal sections of the flat-faced fruit-eating bat at rostral **(A)**, middle **(B)** and caudal **(C)** levels, illustrating the distribution pattern of retinal projections in the ipsi and contralateral sides. The boxed areas in **(A–C)** are shown in high magnification in **(D–F)**, respectively, illustrating the detailed morphology of the retinal axons in the IGL showing cholera toxin-labeled varicosities in the simple endings **(D)**, string-like terminals **(E)** and R2-like terminals **(F)**. Black arrowheads point to sort of fibers. **(G–I)** Camera-lucida drawings that depict the morphology of simple endings **(G)**; string-like terminals **(H)** and R2-like terminals **(I)**. Scale bar 500 μm **(A–C)** and 10 μm **(D–I)**.

**FIGURE 8:**
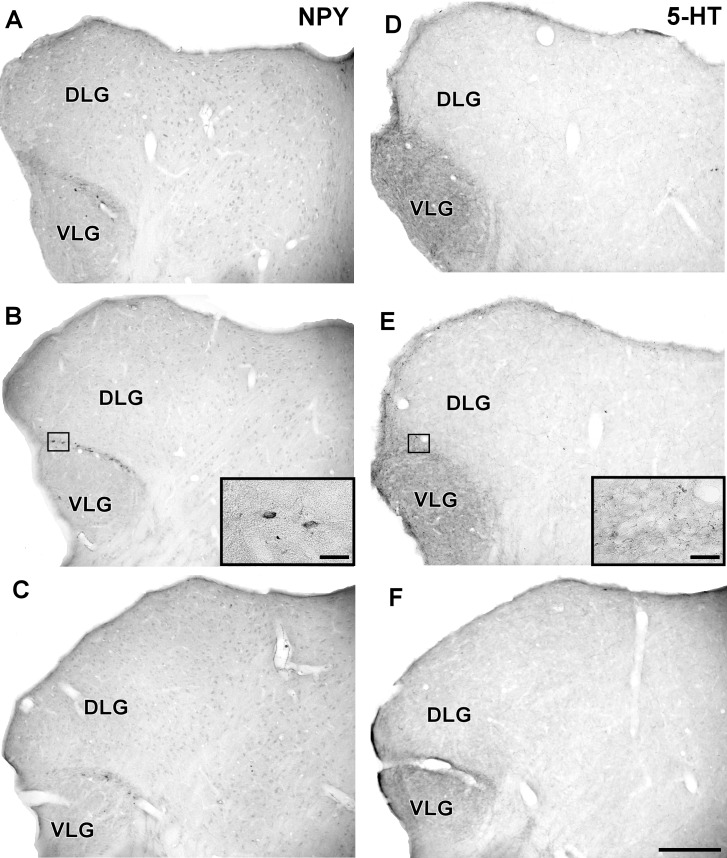
Photomicrographs of the brain sections of flat-faced fruit-eating bat showing the IGL in bright field. **(A–C)** Immunoreactivity against NPY in the IGL at rostral, middle, and caudal levels, respectively. **(D–F)** Immunoreactivity against 5-HT in the IGL at rostral, middle, and caudal levels, respectively. The magnification of the boxed delimited area is shown in detail in the upper-right corner of each respective picture. DLG, dorsal lateral geniculate nucleus; VLG, ventral lateral geniculate nucleus. Scale bar 500 μm.

CTb-IR fibers were visualized bilaterally in all divisions of the lateral geniculate complex, including the IGL (**Figure 7**). A contralateral predominance of this projection was observed in the DLG and VLG. No significant difference (*p* = 0.28) was detected between the ipsilateral (0.0105 ± 0.1801) and contralateral (0.0428 ± 0.0347) sides of the IGL. The distribution of the CTb-retinal terminals made it possible to identify the classical morphology of the IGL as a thin leaflet interposed between the DLG and VLG, at the rostral and middle levels. At the caudal level, the IGL exhibits a descending portion that outlines the VLG medially (**Figure 8C**). As described for the SCN, the optic terminals in the IGL were identified as simple *en passant* varicosities and terminal swellings (**Figures 7D,G**), string-like complexes (**Figures 7E,H**) and type R2-like terminals (**Figures 7F,I**).

#### Neurochemical Characteristics

**FIGURE 9:**
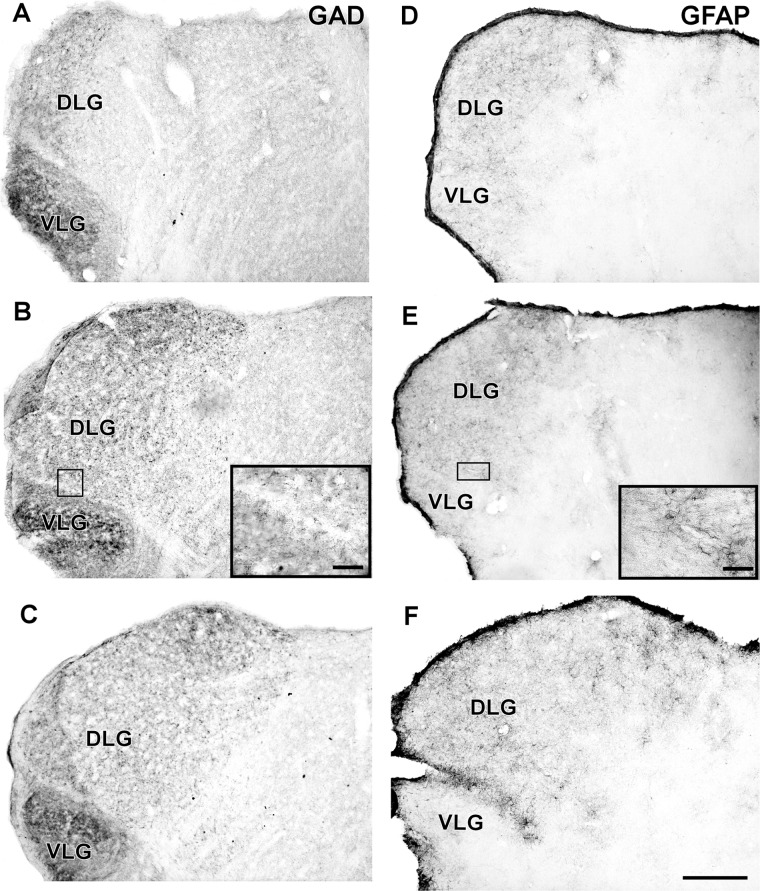
Photomicrographs of the brain sections of flat-faced fruit-eating bat showing the IGL in bright field. **(A–C)** Immunoreactivity against GAD in the IGL at rostral, middle, and caudal levels, respectively. **(D–F)** Immunoreactivity against GFAP in the IGL at rostral, middle, and caudal levels, respectively. The magnification of the boxed delimited area is shown in detail in the upper-right corner of each respective picture. DLG, dorsal lateral geniculate nucleus; VLG, ventral lateral geniculate nucleus. Scale bar 500 μm.

In the IGL of the flat-faced fruit-eating bat, NPY-IR immunoreactivity was detected along the rostrocaudal axis. There were elongated labeled neurons immersed in a sparse plexus of NPY-IR fibers/terminals. NPY-IR elements were not detected in the DLG and VLG (**Figures 8A–C**). A dense plexus of serotonergic fibers/terminals was visualized in the IGL and VLG, in contrast to the DLG, in which 5-HT-IR elements were not detected (**Figures 8D–F**). Immunostaining for GAD was detected throughout the rostrocaudal extent of the thalamic lateral geniculate complex. An intense network of GAD/GABA-IR fibers/terminal was visualized in the IGL, DLG, and VLG (**Figures 9A–C**). GFAP-IR was visualized across the rostrocaudal axis of the IGL. GFAP-IR was also identified in the DLG and VLG. This staining provides evidence of the astrocytic activity of these structures (**Figures 9D–F**).

## Discussion

### Suprachiasmatic Nucleus

The SCN of the flat-faced fruit-eating bat, as in other mammals, is distinguished from the adjacent hypothalamus by the presence of a darkly Nissl-stained cell group located on each side of the third ventricle and dorsal to the optic chiasm. Morphologically, this nucleus exhibits a shape similar to that found in rock cavy (*Kerodon rupestris*) (Nascimento et al., 2010) in that this structure in both animals has a triangular sectional shape at the rostral level and a pear-shaped contour at the middle and caudal levels. The diencephalic topography conforms to the pattern described for all mammals studied (Cassone et al., 1988; Cohen et al., 2010). This is consistent with the suggestion that the mammalian SCN is a phylogenetically conserved neural structure. With respect to SCN volume, few studies have stereological data for this parameter, which makes it difficult to compare species. In our study, the SCN of the flat-faced fruit-eating bat has an estimated mean volume of 0.1041 mm^3^. The average volume in this bat is greater than those observed in rodents, but smaller than those reported for sheep, capuchin monkey, and human, as seen in our findings and in reviewed data (**Table 2**).

According to our results, the SCN of the flat-faced fruit-eating bat receives a bilateral retinal innervation. The application of OD analysis indicates a possible bilateral symmetry in the prevalence of this projection. This pattern is similar to that observed in the few species studied (Supplementary Table S1). In contrast to the big brown bat (*Eptesicus fuscus*) and the Jamaican fruit-eating bat (*A. jamaicense*) (Cotter, 1985), contralateral projections were described in the SCN. An ipsilateral predominance was observed in the Nile grass rat (Negroni et al., 2003) and African mole rat (*Cryptomys anselli*) (Nemec et al., 2004).

A common characteristic of the SCN in all mammalian species studied is bilateral innervation from the retina, except for the golden-mantled ground squirrel (*Spermophilus lateralis*), whose innervation is described to be exclusively contralateral (Smale et al., 1991). The pattern of the retino-SCN innervation has been described as predominantly or almost completely contralateral, predominantly ipsilateral, or with almost complete bilateral symmetry (see comments in Costa et al., 1999).

The RHT is formed by fibers from non-image forming melanopsin-expressing ipRGC and transmits environmental luminance levels to SCN (Dibner et al., 2010; Albrecht, 2012). The monosynaptic RHT fibers terminate directly on VIPergic neurons in the core/ventrolateral portion of the SCN (Ibata et al., 1989; Tanaka et al., 1993). Early studies have established that RHT, is both necessary and sufficient for photic entrainment of the SCN (Morin, 1994). Ablation of all other visual pathways does not alter stable entrainment (Klein and Moore, 1979) and selective transection of the RHT abolishes entrainment with persistence of free-running locomotor rhythms (Johnson et al., 1988). The functional significance of variability in the pattern of RHT innervation in the SCN remains unknown. In an early study (Magnin et al., 1989) an evolutive theory was proposed. It was suggested that the retino-SCN innervation has evolved from a contralateral predominance or bilateral equivalence in rodents to an ipsilateral predominance in insectivores and primates (Magnin et al., 1989). However, there are many conflicting findings around this theory. For example, the retino-SCN projection is described to be ipsilateral in some primate species, such as the chimpanzee (*Pan troglodytes*) (Tigges et al., 1977), bush baby (*Galago alleni*, *Galago demidovii*), potto (*Perodicticus potto*), gibbon (*Hylobates concolor*) and macaque (*Macaca fascicularis*) (Magnin et al., 1989). However, that projection exhibits a contralateral pattern in other primate species, such as Rhesus monkey (Hendrickson et al., 1972; Moore, 1973), squirrel monkey (Tigges and O’Steen, 1974) and common marmoset (Costa et al., 1999; see comments in Costa et al., 1999).

Morphological and electrophysiological analyses suggest at least five types of ipRGCs projecting predominantly to brain regions involved in non-image forming visual function, as well as projections to brain regions important for image formation (Schmidt et al., 2011). Interestingly, Baver et al. (2008) have demonstrated two types of ipRGCs in the mouse that project to the SCN by injection of a virus into the SCN, followed by *trans*-synaptic infection of the retinal terminals and retrograde transport to both retinas. Despite that, the specific terminal fields of these types of ipRGCs in the SCN remain unknown. The present results in the flat-faced fruit-eating bat have shown three distinct terminal morphologies in the SCN, suggesting different wiring configurations of the retinal fibers in the SCN. Given these findings, we believe that the intraocularly injected CTb can provide clues to clarify differential ipRGC innervation in the SCN. Furthermore, the morphological diversity of the endings suggests a differential effect on postsynaptic targets (Petrof and Sherman, 2013), as well as a functional division in the SCN. Taken together, the present results in the flat-faced fruit-eating bat reveals the RHT with extensive and morphological distinct terminals within the SCN, reinforcing the key role of the RHT in conveying photic cues necessary for circadian rhythms entrainment.

With respect to neuropeptidergic phenotype, VP-IR neurons are present in the dorsomedial part of the SCN in the flat-faced fruit-eating bat. A similar pattern is described in other mammalian species studied (Supplementary Table S1). On the other hand, in the Japanese long-fingered bat (*Miniopterus schreibersii*) (Mikami et al., 1988) and mustached bat (*Pteronotus parnellii*) (Rao and Kanwal, 2004), the topography and description of VP cells in the SCN were not very precise. At present, in musk shrew (Tokunaga et al., 1992), mink (*Mustela vison*) (Martinet et al., 1995) and eusocial naked mole rat (*Heterocephalus glaber*) (Rosen et al., 2008), VP-IR cells have not been identified in the SCN probable due any circadian effect. It is possible for VP and GFAP that the immunohistochemical signal decrease according to the time of the day. VP mediated by V1a receptor enhances the amplitude of firing rates in the SCN during subjective day (Mihai et al., 1994) and increase the SCN output (Ingram et al., 1996; Ingram et al., 1998). Furthermore, Li et al. (2009) demonstrated that VP/V1a regulates the expression of CCG in the SCN. They have shown that VP/V1a positively regulate the transcription of a CCG output gene PK2 in V1a knockout mice. When VP levels are high, these animals displayed reduced PK2 mRNA levels during the subjective day (Li et al., 2009). Together, these data suggest that VP play a critical role in the amplification and synchronization of the endogenous rhythmicity of SCN, as well as in the distribution of circadian signals (Kalsbeek et al., 2010). For the flat-faced fruit-eating bat, a possible role of these cells population in their circadian activity pattern of the SCN cannot be excluded.

The predominant distribution of VIP-IR cells in the ventromedial SCN of the flat-faced fruit-eating bat is similar to that observed in two species of rodents (Supplementary Table S1). In contrast, in Virginia opossum (*Didelphis virginiana*), gray short-tailed opossum (*Monodelphis domestica*) (Cassone et al., 1988), and marmoset (Costa et al., 1998), VIP-IR neurons are localized in the dorsomedial portion of the SCN. A ventrolateral predominance was reported in mouse (Abrahamson and Moore, 2001; Morin et al., 2006), Nile grass rat (Smale and Boverhof, 1999), blind mole rat (Negroni et al., 1997) and capuchin monkey (*Sapajus apella*) (Rocha et al., 2014). VIP-IR cells and fibers were described in the little brown bat SCN (Laemle and Cotter, 1988). Functional studies have shown that most afferents to the SCN, such as glutamatergic retinal afferents, NPY fibers from the IGL and 5-HT fibers from the raphe, make synaptic contacts with VIP cells (Hisano et al., 1987, 1988; Ibata et al., 1989). Besides that, it was evidenced that VIP content in the rat SCN does not show circadian rhythm in constant darkness, but under light–dark conditions, it decreases over the course of the light phase, recovering gradually during the dark phase (Shinohara et al., 1993). Taken together, these data corroborate the hypothesis that VIP neurons are involved in the synchronization of circadian rhythms. A more recent study provides evidence supporting a role in promoting rhythmicity for VIP neurons (Aton et al., 2005). VIP neurons participate in local connections within the ventral region and with VP-IR neurons in the dorsal region (Ibata et al., 1993). Furthermore, VP cells project to the VIP cells, providing an anatomical support for a feedback mechanism, through which messages related to environmental lighting conditions may be constantly modulated by temporal information on a circadian basis (Jacomy et al., 1999). The VIPergic mechanisms remain to be investigated in the flat-faced fruit-eating bat.

In contrast to the gray mouse lemur (*Microcebus murinus*) (Bons et al., 1990), humans (Mai et al., 1991), and the lesser hedgehog tenrec (*Echinops telfairi*) (Künzle and Unger, 1992), NPY-ergic neurons were not identified in the SCN of the flat-faced fruit-eating bat. In fact, a plexus of NPY-IR fibers/terminals was visualized in its ventrolateral portion in the current study. This configuration was observed in previous studies (Supplementary Table S1). However, a distinctive pattern was detected in hamster (Card and Moore, 1984) and mouse (Cassone et al., 1988; Abrahamson and Moore, 2001). NPY-IR fibers were absent in the little brown bat SCN (*Myotis lucifugus*) (Laemle and Cotter, 1992). It is postulated that NPY is involved in the modulation of SCN activity in response to photic and non-photic stimuli via GHT (Muscat and Morin, 2006).

The present study shows a dense plexus of serotonergic fibers, predominantly in the ventral part of the SCN, of the flat-faced fruit-eating bat. This characteristic is similar to what is described for several mammalian species (Supplementary Table S1). In rodents, serotonergic projections to the SCN originate in the median raphe nucleus (Moga and Moore, 1997; Morin, 1999; Hay-Schmidt et al., 2003). However, in the flat-faced fruit-eating bat, the identification of 5-HT-IR neurons in the midbrain raphe nuclei combined with hodological techniques would be needed to draw conclusions regarding the origin of fibers in the SCN.

The GAD-IR fibers were detected in the SCN of the flat-faced fruit-eating bat, similar to what has been described in the golden hamster (Morin et al., 1992) (Supplementary Table S1). On the other hand, our data differ from results obtained in rodents, in which GAD-IR neurons were found in the SCN (Card and Moore, 1984; Moore and Speh, 1993; Abrahamson and Moore, 2001) with different degrees of co-localization with VP and VIP (Buijs et al., 1995; Tanaka et al., 1997; Castel and Morris, 2000). The role of GABA in the SCN is still not well understood. It is suggested that GABA controls the amplitude of circadian rhythms in SCN neurons (Aton et al., 2006) and facilitates communication between SCN regions during the propagation of photic input (Han et al., 2012). Furthermore, there is evidence that this neurotransmitter is able to synchronize clock cells within the SCN (Liu and Reppert, 2000).

The SCN in the flat-faced fruit-eating bat is marked by dense GFAP-IR cells as compared to adjacent hypothalamic areas. Our results are similar to those in other mammalian species studied (Supplementary Table S1). However, our results differ from findings in the rock cavy SCN, in which the GFAP-IR cells were detected with the same density as those in surrounding hypothalamic areas (Nascimento et al., 2010). This protein was also observed in marmosets (Engelberth et al., 2014) and humans (Stopa et al., 1999). In rodents, such as the rat (Hajós, 2008), hamster (Lavialle and Servière, 1993; Lavialle et al., 2001) and mouse (Santos et al., 2005), GFAP expression exhibits a circadian rhythm.

### Intergeniculate Leaflet

As in most mammals, the IGL in the flat-faced fruit-eating bat was identified as a thin leaflet interposed between the DLG and VLG. This structure is delimited by retinal projections and neuropeptidergic phenotype. Data related to the volume and morphometric features of the IGL are scarce in the scientific literature. This fact, limits a general comparison among species (**Table 2**). The stereological analysis revealed that flat-faced fruit-eating bat IGL has an estimated mean volume of 0.0378 mm^3^.

The IGL of the flat-faced fruit-eating bat receives a bilateral retinal projection and the OD analysis indicates possible symmetrical innervation. This configuration was visualized in the short-tailed fruit bat (Scalia et al., 2015) (Supplementary Table S2). Differently from present work, a predominantly contralateral projection was described in Nile grass rat (Negroni et al., 2003), degu (Goel et al., 1999), and California ground squirrel (Major et al., 2003). A predominant ipsilateral innervation was observed in fossorial mole-lemmings (Herbin et al., 1994). Moreover, the IGL of the flat-faced fruit-eating bat exhibits the same type of retino-SCN terminals, simple *en passant*, string-like configurations and type R2-like terminals suggesting functional subdivision in the nucleus.

In the IGL of the flat-faced fruit-eating bat, NPY-IR scattered neurons and a network of stained fibers/terminals were distributed throughout the rostrocaudal extension (Moore and Card, 1994). This result was also found in two species of rodents (Supplementary Table S2). In rat (Morin et al., 1992), hamster (Moore and Card, 1994), Nile grass rat (Smale and Boverhof, 1999), and degu (Goel et al., 1999), the IGL is easily identified by its abundant expression of NPY-IR cells and fibers. NPY-positive perikarya were not identified in little brown bat (Laemle and Cotter, 1992). The presence of NPY in the IGL of the flat-faced fruit-eating bat, although small in quantity, as well as fibers/terminals in the SCN, suggests the existence of the GHT in this species.

5-HT-IR cells were not identified in the IGL of the flat-faced fruit-eating bat, although a dense plexus of serotonergic fibers/terminals were visualized throughout this structure. This pattern was also described in other species of mammalians (Supplementary Table S2). In contrast to Nile grass rat, moderate plexus of 5-HT-IR was reported in IGL (Smale and Boverhof, 1999).

A plexus of GAD-IR fibers/terminals was detected in the IGL of the flat-faced fruit-eating bat. A similar IR pattern was reported in the golden hamster (Morin et al., 1992). In addition, GAD-IR neurons were visualized in the rat (Moore and Speh, 1993; Moore and Card, 1994), ground squirrel (Agarwala et al., 1992) and primate PGN, such as the marmoset (Lima et al., 2012), Rhesus monkey and Cynomolgus monkey (Livingston and Mustari, 2000). In the flat-faced fruit-eating bat, neither SCN or IGL have shown GAD-IR cells, despite a plexus of GAD-IR fibers have been revealed throughout rostrocaudal length. Interestingly, for some unknown reason, other places GABA-contained cells (e.g., the raphe nuclei) could project to the circadian centers in this species to modulate circadian rhythmicity.

Glial fibrillary acidic protein is present throughout the rostrocaudal extent of the flat-faced fruit-eating bat IGL. Our results agree with those described in previous studies (Supplementary Table S2). A variation in circadian expression of GFAP in the IGL has been demonstrated for the mouse (Moriya et al., 2000). However, the role of GFAP in the IGL related to circadian rhythm regulation is not well understood.

## Conclusion

The present work provides the characterization of retinal innervation and neurochemical signature of the SCN and IGL in a South America endemic chiropteran, the flat-faced fruit-eating bat. The SCN core/ventrolateral and shell/dorsomedial organization have shown differences in this bat. Interestingly, GAD-IR is present as a plexus of axons in the SCN as well as in the IGL which probably has its origins in cells of the midbrain raphe nuclei. Furthermore, the morphological analysis of the retinal fibers has suggested a functional subdivision inside the SCN in the flat-faced fruit-eating bat. Obviously, additional hodological, electrophysiological, behavioral and gene expression studies are needed to establish a coherent functional division of the SCN for this species. In addition, this study is the first to describe the morphological diversity of retinal fibers inside the SCN and IGL and to provide the neuroanatomical bases of the structures involved in the control and modulation of circadian rhythms in a chiropteran species using OD and stereological methods. In more general terms, the differences found in the circadian centers of the flat-faced fruit-eating bat may be related to the differences in the nocturnality pattern described in this species.

## Author Contributions

NS, MC, and EN designed the research, analyzed data, and wrote the manuscript. LS, FL, HM, JSC, RE, and RL performed the research and analyzed the data. JCC revised the paper critically. MB, MS, and PM participated in the analysis of the results. EN designed, supervised studies, interpreted results, and prepared the manuscript.

## Conflict of Interest Statement

The authors declare that the research was conducted in the absence of any commercial or financial relationships that could be construed as a potential conflict of interest.

## Abbreviations

5-HT: serotonin
CCG: clock-controlled genes
CTb: cholera toxin subunit B
CTS: circadian timing system
DLG: dorsal lateral geniculate nucleus
GAD: glutamic acid decarboxylase
GFAP: glial fibrillary acidic protein
IGL: intergeniculate leaflet
ipRGC: intrinsically photosensitive retinal ganglion cells
IR: immunoreactive
NPY: neuropeptide Y
PK2: prokineticin 2
SCN: suprachiasmatic nucleus
VIP: vasoactive intestinal peptide
VLG: ventral lateral geniculate nucleus
