# Supplementary material for: The Suprachiasmatic Nucleus and the Intergeniculate Leaflet of the Flat-Faced Fruit-Eating Bat (Artibeus planirostris): Retinal Projections and Neurochemical Anatomy
Source: Front Neuroanat. 2018 May 15;12:36. doi: 10.3389/fnana.2018.00036 (PMC5962671; doi:10.3389/fnana.2018.00036)
Supplement: Supplementary file 1 [file Table_1.pdf]

Supplementary Material

Article Title: The suprachiasmatic nucleus and the intergeniculate leaflet of the flat-faced fruit-eating bat (*Artibeus planirostris*): retinal projections and neurochemical anatomy

Nelyane Nayara.M. Santana, Marília A. S. Barros , Helder H. A. Medeiros, Melquisedec A. D. Santana, Lara Laise Silva, Paulo Leonardo A. G. Morais, Fernando Vagner L. Ladd, Jeferson S. Cavalcante, Ruthnaldo R. M. Lima, Judney Cley Cavalcante, Miriam Stela M. O. Costa, Rovena Clara J. G. Engelberth, Expedito S. Nascimento Jr*

* Correspondence: expeditojr@cb.ufrn.br

Supplementary Table S1: Comparative analysis of the retino-SCN innervations in mammals and similarities on neurochemical content in the SCN of the *Artibeus planirostris* and other animals.

**Animals and References**	***Pattern of retinal projection***			***Neurochemical pattern in SCN***		
	Predominantly contralateral	***Predominantly ipsilateral***	***Symmetrical predominance***	***Neurochemical characteristics of the SCN in the Artibeus planirostris***	***Similar pattern***	***References***
California ground squirrel/Major et al., 2003	■			**VP-IR cells in dorsomedial portion**	Rat	Van den Pol and Tsujimoto, 1985<br>Buijs et al., 1995<br>Moore et al., 2002<br>Morin et al., 2006
Rat/Johson et al., 1988<br>Levine et al., 1991	■					
Guinea pig/Cassone et al., 1988	■					
Mouse/Scalia et al., 2015			■		Mouse	Abrahamson and Moore, 2001
Short-tailed fruit bat/Scalia et al., 2015			■		Tree shrew (*Tupaia belangeri chinensis*)	Ni et al., 2014
Egyptian fruit bat/Magnin et al., 1989			■			
Artibeus planirostris, present study			■		Ground squirrel	Reuss et al., 1989
Big brown bat (*Eptesicus fuscus*)/Cotter, 1985	■				Japanese horseshoe Bat (*Rhinolophus ferrumequinum*)	Kumamoto et al., 1992
Jamaican fruit-eating bat (*Artibeus jamaicenses*)/Cotter, 1985	■			**VIP-IR cells in ventromedial portion**	Degu (*Octodon degus*)	Goel et al., 1999
Short-tailed fruit bat/Scalia et al., 2015			■		Rock cavy	Nascimento Jr et al., 2010

Rock cavy/Nascimento Jr et al., 2010	■					
*Nile grass rat/Smale and Boverhof, 1999		■		**NPY-IR fibers/terminals in ventrolateral portion**	Ground squirrel	Reuss et al., 1989
Blind mole rat (*Spalax ehrenbergi*)/Negroni et al., 1997	■	■			Common mole rat (*Cryptomys hottentotus*)	Negroni et al., 2003
African mole rat (*Cryptomys anselli*)/Nemec et al., 2004		■			Degu	Goel et al., 1999
House musk shrew (*Suncus murinus*)/Mizuno et al., 1991		■				

Animals and References	***Pattern of retinal projection***			***Neurochemical pattern in SCN***		
	Predominantly contralateral	***Predominantly ipsilateral***	***Symmetrical predominance***	***Neurochemical characteristics of the SCN in the Artibeus planirostris***	***Similar pattern***	***References***
Sheep/Tenosseaud et al., 1994			■	**5-HT-IR fibers/terminals in ventral portion**	Rat<br>Hamster<br>Cat	Ueda et al., 1983
Black gibbon/Magnin et al., 1989		■			Mouse	Abrahamson and Moore, 2001
Marmoset/Costa et al., 1999	■				Degu	Goel eta l., 1999
Capuchin monkey/Pinato et al., 2009	■				Blind mole rat	Negroni et al., 1997
Cynomologus monkey (*Macaca fasciculares*)/Magnin et al., 1989		■			Nile grass rat	Smale and Boverhof, 1999
					Ground squirrel	Smale et al., 1991
					Cairo spiny mouse (*Acomys cahirinus*)<br>Golden spiny mouse (*Acomys russatus*)	Cohen et al., 2010
				**GABA-IR fibers/terminals**	Golden hamster	Morin et al., 1992
				**Intense GFAP**	Rat<br>Hamster	Morin et al., 1989
					Ground squirrel	Smale et al, 1991
					Nile grass rat	Smale and Boverhof, 1999
