# Supplementary material for: The Suprachiasmatic Nucleus and the Intergeniculate Leaflet of the Flat-Faced Fruit-Eating Bat (Artibeus planirostris): Retinal Projections and Neurochemical Anatomy
Source: Front Neuroanat. 2018 May 15;12:36. doi: 10.3389/fnana.2018.00036 (PMC5962671; doi:10.3389/fnana.2018.00036)
Supplement: Supplementary file 2 [file Table_2.pdf]

Supplementary Material

Article Title: **The suprachiasmatic nucleus and the intergeniculate leaflet of the flat-faced fruit-eating bat (*Artibeus planirostris*): retinal projections and neurochemical anatomy**

Nelyane Nayara.M. Santana, Marília A. S. Barros , Helder H. A. Medeiros, Melquisedec A. D. Santana, Lara Laise Silva, Paulo Leonardo A. G. Morais, Fernando Vagner L. Ladd, Jeferson S. Cavalcante, Ruthnaldo R. M. Lima, Judney Cley Cavalcante, Miriam Stela M. O. Costa, Rovena Clara J. G. Engelberth, Expedito S. Nascimento Jr*

* Correspondence: expeditojr@cb.ufrn.br

Supplementary Table S2: Comparative analysis of the retino-IGL innervation in mammals and similarities on neurochemical content in the IGL of the Flat-faced fruit-eating bat and other animals.

**Animals**	***Pattern of retinal projection***			***References***
	Predominantly contralateral	Predominantly ipsilateral	Symmetrical predominance	
California ground squirrel				Major et al., 2003
*Ground squirrel (*Citellus tridecemlineatus*)				Agarwala et al., 1989
*Gold-mantle ground squirrel				Smale et al., 1991
Degu				Goel et al., 1999
*Golden Hamster				Morin et al., 1992
*Rock cavy				Nascimento Jr et al., 2010
Nile grass rat				Smale and Boverhof, 1999
African mole rat				Nemec et al., 2004
House musk shrew				Mizuno et al., 1991
Fossorial mole-lemmings (*Ellobius talpinus*) (*Ellobius lutescens*)				Herbin et al., 1994

Neurochemical characteristics Flat-faced fruit-eating bat IGL	***Similar pattern***	***References***
**Scattered NPY-IR cells and fibers/terminals**	Rock cavy	Nascimento Jr et al., 2010
	Common mole rat	Negroni et al., 2003
	[1]Capuchin monkey	Pinato et al., 2009
	[1]Marmoset	Costa et al., 1998<br>Chevassus-au-Louis and Cooper, 1998<br>Lima et al., 2012
	[1]Gray mouse lemur	Chevassus-au-Louis and Cooper, 1998
	[1]Cynomologus monkey	Chevassus-au-Louis and Cooper, 1998
	[1]Humans	Moore, 1989.
**5-HT-IR fibers/terminals**	Rat	Mantyh and Kemp, 1983
	Hamster	Morin et al., 1992
	[1]Marmoset	Lima et al., 2012
	[1]Capuchin monkey	Pinato et al., 2009
**GABA-IR fibers/terminals**	Golden hamster	Morin et al., 1992
**GFAP-IR**	Nile grass rat	Smale and Boverhof, 1999
	Rat	Morin et al., 1989
	Hamster	Morin et al., 1989
	Gold-mantle ground	Smale et al., 1991

Short-tailed fruit bat		Scalia et al., 2015
Flat-faced fruit-eating bat		Present study

squirrel	
Rock cavy	Nascimento Jr et al., 2010
Mouse	Santos et al., 2005

* discrete contralateral predominance

[1] The neuroactive substances was observed in pregeniculate nucleus, primate homologus of the IGL.
